# Relationship between Implant Length and Implant Stability of Single-Implant Restorations: A 12-Month Follow-Up Clinical Study

**DOI:** 10.3390/medicina56060263

**Published:** 2020-05-27

**Authors:** Juan Manuel Aragoneses, Javier Aragoneses, Vanessa Arlette Brugal, Margarita Gomez, Ana Suarez

**Affiliations:** 1 Department of Dental Research, Federico Henriquez y Carvajal University, Santo Domingo 10106, Dominican Republic; jmaragoneses@gmail.com; 2 Department of Dentistry, Federico Henriquez y Carvajal University, Santo Domingo 10106, Dominican Republic; javias511@gmail.com; 3 Department of Pediatric Dentistry and Orthopedics, School of Dentistry, Universidad Autónoma de Santo Domingo, Santo Domingo 10105, Dominican Republic; prasega@hotmail.com; 4 Department of Preclinical Dentistry, School of Biomedical Sciences, Universidad Europea de Madrid, 28108 Madrid, Spain; margarita.gomez2@universidadeuropea.es

**Keywords:** dental implant, implant length, primary stability, resonance frequency analysis, secondary stability

## Abstract

*Background and Objectives*: Implant stability in vivo is contingent on multiple factors, such as bone structure, instrument positioning and implant surface modifications, implant diameter, and implant length. Resonance-frequency analysis is considered a non-invasive, reliable, predictable, and objective method by which to evaluate implant stability, due to its correlation with bone-to-implant contact. The purpose of this study was to evaluate the effect of implant length on the primary and secondary stability of single-implant crown rehabilitations, as measured by resonance-frequency analysis at different times. *Materials and Methods*: Implants of 10 and 11.5 mm were placed, and the resonance frequency was measured at the time of surgery (T0), as well as at 3 (T1), 6 (T2), and 12 (T3) months post-surgery. *Results*: A total of 559 implants were placed in 195 patients. Significant differences were observed when comparing the implant stability quotient (ISQ) values at T1, with values for 10-mm implants being greater than those for 11.5-mm implants (*p* = 0.035). These differences were also observed when comparing ISQ values for buccal and lingual areas. At T0, T2, and T3, no significant differences in ISQ values were observed. The use of 10-mm implants in the anterior maxilla yielded significantly greater values at T0 (*p* = 0.018) and T1 (*p* = 0.031) when compared with 11.5-mm implants. Significant differences in measurements were observed only for buccal areas (*p* = 0.005; *p* = 0.018). When comparing the sample lengths and sex, women with 11.5-mm implants showed significantly lower results than those with 10-mm implants (*p* < 0.001). *Conclusions*: There is a direct relationship between implants of a smaller length and greater ISQ values, with this relationship being most evident in the maxilla and in women.

## 1. Introduction

Primary (or mechanical) stability (PS) following mechanical implant insertion is classified as the absence of axial, lateral, and rotational micro-movements (or, in other words, biometric stability between the bone and the implant), and has been described as a fundamental requirement for achieving osseointegration [1].

In animal experiments, the scientific literature details that once an implant has been inserted into the bone tissue, the said implant presents signs of so-called mechanical stability. From this point on—between one and two weeks following implant placement—the remodeling processes of the peripheral bone begins and continues until osseointegration is achieved [2]. At this point, the implant begins to demonstrate signs of secondary or biological stability (SS), which, in turn, alters stability values compared to those obtained in the primary stage [3].

Resonance-frequency analysis (RFA) is considered to be a non-invasive, reliable, predictable, and objective method by which to evaluate implant stability, due to its correlation with bone-to-implant contact (BIC). Devices using this principle consist of specific electromechanically stimulated transducers screwed into the implant. Depending on the vibration of the implant–transducer interface, the device yields a numerical value expressed as a quotient, known as the implant stability quotient (ISQ), with a range that oscillates between 1 and 100, with 100 being the value corresponding to the maximum vibration [4]. Following implant insertion and during the first 12 weeks, biological changes happen at the bone–implant interface level; infiltration of inflammatory and mesenchymal cells in the implant chamber leads to a series of parallel bone apposition and resorption events, which, in turn, lead to the formation of new mineralized tissue [2]. The cited changes produce an alteration of the BIC level, which, in turn, translates into variations in the natural vibration of the transducer due to variation in the stiffness of the complex, resulting in varied ISQ values [5]. As such, measuring the rigidity of BIC with this method can provide reliable objective data about the level of implant osseointegration [5], albeit within certain limits, and can significantly predict implant survival when applied after the healing period [6].

Different studies have confirmed that implant stability is contingent on multiple factors in vivo, such as bone structure, instrument positioning and implant surface modifications, implant diameter, and implant length [7]. The use of the latter factor should be based on biomechanical considerations [8] and long-term prognosis, as some studies have shown a correlation between implant survival and length over different time frames and in different locations, which, in turn, could indicate the necessity of invasive bone augmentation procedures when longer lengths are used [9]. Nonetheless, when evaluating the possible in vivo relationship between implant stability using RFA and implant length, prospective clinical studies comparing the relationship between the two variables for single-implant crown rehabilitations before and after implant loading could not be found. Furthermore, there have been contradictory and insufficient results presented among existing studies in this area; Sim and Lang [10] stated than an implant of a longer length is associated with greater PS, while others have reported the opposite relationship between these variables [11,12] or no correlation at all [13,14,15,16,17]. Similarly, discrepancies have been found when analyzing the relationship between SS and implant length, as a number of studies have shown contradictory results [13,14,15,18].

Due to this high discrepancy among studies, the objective of the present study was to determine whether the length of an implant affects the primary and secondary stability of single-implant crown rehabilitation measurements using the RFA system and to verify the relationship between these values and with other individual factors, like sex and the location of the implants.

## 2. Materials and Methods

### 2.1. Study Design

A prospective study was conducted at Federico Henriquez y Carvajal University clinic between 2016 and 2018. All selected patients signed an informed consent prior to participating in the clinical study and were informed about the necessary interventions following the ethics committee’s approval (Approval number: 10/2010).

### 2.2. Implant Characteristics

**Figure 1:**
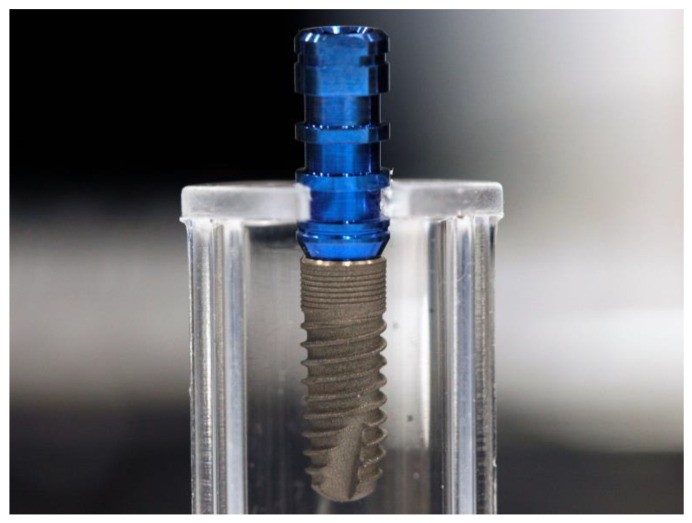
Model of the implant used for the study. The sole variations were in length and diameter.

Zinic^®^ (Ziacom Medical, SL. Madrid, Spain) titanium grade IV implants were used in this study. The body presented active spirals of a reduced angle, double spiral, and cross-sectional apical windows, and an atraumatic apex. Furthermore, the implants presented an internal hexagonal connection, a conical bezel, and a platform switch. All implants studied received the same surface treatment and, as such, the same topography (Figure 1). The sole variations were in length (10 and 11.5 mm) and diameter (3.7, 4.0, and 4.3 mm).

### 2.3. Study Population

The inclusion criteria for the selection of patients were as follows: Partially edentulous ASA I (physical status given by the American Society of Anesthesiologists to normal healthy non-smoker subjects with minimal or non-existent alcohol use) patients who required a single-implant crown rehabilitation and had sufficient bone height to accommodate an implant of at least 10 mm in length. To ensure this, panoramic radiography evaluation prior to the surgical phase was performed by the operator. The exclusion criteria were as follows: Smokers, previous treatment with bisphosphonates, previous chemotherapy or radiotherapy treatment, active periodontal disease, diabetics, bruxism sufferers, and poor oral hygiene.

### 2.4. Clinical Steps

The surgical phase (T0) corresponded to the insertion of the implant. The following guidelines were respected at all times: The anesthetic technique was local and infiltrative; the selected anesthetic was articaine with epinephrine at a concentration of 1:200,000. An incision was made, and the mucosa detached until the bone tissue was revealed. The implants were inserted as per the manufacturer’s instructions. The implants were inserted into the bone tissue at a counter-angle with a minimum insertion torque of 35 N/cm. The final insertion of the implant was carried out manually with a ratchet. The resonance-frequency analysis (RFA) was conducted using the Osstell Mentor^®^ for both buccal and lingual areas, and, prior to this, a transducer or SmartPeg^®^ was screwed into the head of the implant. To obtain ISQ values, measurements were carried out with two different transducers for each implant. In order for these measurements to be considered valid, there could not be a variation of more than 2 ISQ units for each measurement between the two transducers. In the event of a discrepancy of less than 2 units for each measurement between the two transducers, the average of both measurements was used. The closing cap was placed accordingly. The site was stitched up with 3/0 thread. Following the surgery, each patient was given 1 mL of betamethasone sodium phosphate/betamethasone acetate in a sterile aqueous suspension, intramuscularly, which corresponded to 6 mg. The participants were also treated with 875 mg of amoxicillin and 125 mg of clavulanic acid every 12 h for five days starting from the day before the surgery, as well as 600 mg of ibuprofen with arginine every 12 h for three days starting from the day of implant insertion. In the event of pain, 575 mg of magnesium metamizole was also administered. Patients were informed of the techniques required to maintain adequate oral hygiene at the site of the surgery (surgical brush and associated parts) and were told to apply 0.2% chlorhexidine gel every eight hours for 10 days. At Days 7–10, the stitches were removed. In the second phase of surgery (T1), which took place three months after implant placement, the closing cap was removed, the RFA was measured, and the healing abutment was placed. Since measurement of the RFA by Osstell Mentor^®^ had the disadvantage of having to disconnect the prosthetic elements in order to fit the SmartPeg^®^, for the prosthetic rehabilitation, which took place 14 days after T1, single metal-ceramic crowns were fixed with provisional material (Temp–Bond; Kerr, Orange, CA, USA). At six months and one year following implant insertion, the first (T2) and second (T3) control phases took place. The prosthetic rehabilitation was removed, and ISQ values were once again measured in the same way as at T0. All interventions were performed by the same operator. During T1, T2, and T3, all implants that did not survive or that presented a peri-implant site with a pocket depth of ≥5 mm with concomitant bleeding or suppuration on probing and marginal bone loss of ≥2 mm were excluded from the study [19].

### 2.5. Statistical Analysis

Comparisons of average ISQ values were carried out using a two-tailed model, with a null hypothesis defined as equal medians between groups. The most adequate comparison method was the two-sample *t*-test; paired *t*-tests were used to compare ISQ values longitudinally between the baseline (at the time of implant placement) and the subsequent results in the same patient. Comparison of ISQ values between groups was conducted using unpaired *t*-tests. The level of significance was set at α = 0.05.

## 3. Results

In total, 559 endosseous implants were placed in 195 patients. The survival rate was 100%, and no implant was excluded from the study.

Out of all implants, 45.97% were placed in women and 54.03% were placed in men, with the number of implants placed in men being significantly greater than the number placed in women (*p* = 0.007).

When comparing absolute ISQ values, understood as the average of the measurements taken for buccal (B) and lingual (L) areas for the same implant, we did not observe significant differences at T0 for either length.

Significant differences were observed when comparing absolute ISQ values at T1, with values for 10-mm implants being greater than those for 11.5-mm implants. These differences were also observed when comparing ISQ values for B and L (Table 1).

**Figure 2:**
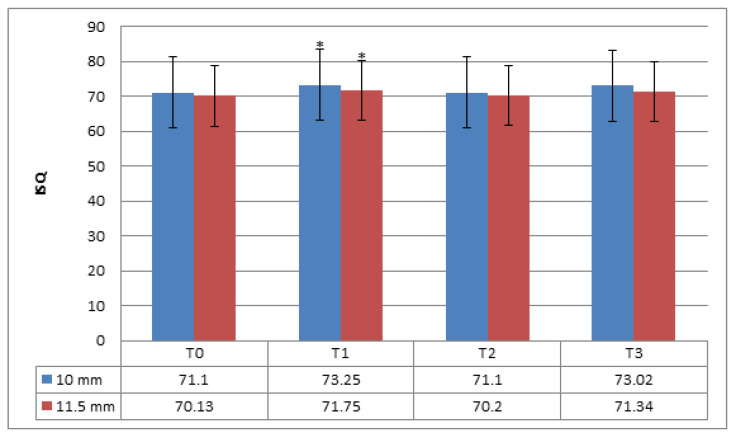
Absolute implant stability quotient (ISQ) values for different lengths measured at follow-up points. * Significant differences were found after three months of implant placement between 10 and 11.5 mm with unpaired *t*-tests (*p* = 0.035) (α = 0.05). T0: at the time of implant placement; T1: 3 months after implant placement; T2: 6 months after implant placement; T3: 12 months after implant placement.

At T2 and T3, no significant differences in ISQ values were observed for either length. However, increases in absolute T1 values occurred, although these later dropped and became similar to those obtained at T0. Implants with a length of 10 mm returned greater values than 11.5-mm implants at each follow-up point (Figure 2).

Statistical differences across the various follow-up points were found for each length (Table 2).

Differences expressed in percentage were also analyzed (Table 3).

When analyzing the lengths and locations of implants at T0, significant differences were found in the anterosuperior area (Table 4). When analyzing the values for B and L, for the same area, also at T0, significant differences in measurements were observed only for B, with the value for 10-mm implants being 73.41 ± 6.96, in comparison to 67.16 ± 8.93 for 11.5-mm implants (*p* = 0.005). At T1, the value for 10-mm implants was 77 ± 8.92, in comparison with 70.95 ± 7.54 for 11.5-mm implants (*p* = 0.018).

No analysis was performed on the effect of different lengths in the anterior area of the mandible due to the small sample size for 10-mm implants (*n* = 1). No significant differences were found for the posterior area of the mandible for either length (Table 5).

When analyzing average ISQ values, understood as the average of the absolute ISQ values obtained for both lengths, the value for the mandible was 71.18 ± 11.08, in comparison with 68.98 ± 10.41 for the maxilla (*p* < 0.001). When assessing the length, we observed statistically significant differences for 10-mm implants placed in the maxilla (ISQ value of 69.96 ± 10.03), in comparison with 11.5-mm implants also placed in the maxilla (ISQ value of 68.50 ± 10.54), resulting in *p* = 0.024. However, no such significant differences were found in the mandible when comparing both lengths.

The average ISQ value for men was 70.99 ± 9.91, whereas for women, it was 69 ± 11.70, with the difference being statistically significant (*p* < 0.001). When comparing the sample lengths and sex, women with 11.5-mm implants were found to have significantly lower results than those with 10-mm implants (*p* < 0.001). When 10- (*p* < 0.001) and 11.5-mm implants (*p* < 0.001) in men were compared (Table 6), their results were also significantly lower.

## 4. Discussion

This study investigated the relationship between stability values measured with RFA and implant length, and correlations with other factors, such as sex and the location of the implants. When analyzing possible differences in terms of sex in the present study, considering average ISQ values, we observed higher values in men with 11.5-mm implants than in women with implants of the same length. As for 10-mm implants, we observed significant differences in favor of men, but only when compared with 11.5-mm implants in women. There were no such differences when comparing to 10-mm implants in women (*p* = 0.434), while 11.5-mm implants in women displayed the lowest stability values.

In a retrospective clinical study using implants of different lengths, Turkyilmaz and McGlumphy [20] obtained significantly higher ISQ values for men compared with women. In the same study, they found higher mean bone density values at implant sites in males than in females, highlighting the significant influence of that parameter on implant stability. Similarly, when assessing PS, Ostman et al. [12] found a lower implant stability in women than in men; however, bone quality could not be clinically measured and, therefore, no association could be established between variables. Furthermore, Guler et al. [16] only found evidence of clinical differences between sexes when the RFA was performed at eight weeks following implant placement but not at the time of surgery or at 12 months after surgery. Nevertheless, none of the aforementioned studies showed correlations between sex and implant length. Therefore, the results obtained in this analysis suggest that when carrying out sex comparisons, implant length is a variable that must be taken into account.

When analyzing the direct relationship between ISQ values and length, this study provided evidence that the highest values are associated with 10-mm implants. However, these differences were only significant at three months and remained that way when breaking down ISQ measurements for buccal and lingual areas. As magnetic RFA evaluates the stiffness of the bone–implant interface in a directional way [21], when statistical differences between lengths were evaluated at each follow-up time, these were expected to be present on both sides, considering that B and L measurements were performed in the same axial direction. Therefore, in this case, differences between lengths and an analysis of the variability pattern over time can be done in terms of absolute values, established as the mean of both measurements. Nonetheless, it is important to notice that single-directional measurements allow the identification of the most and least stable directions. In a study by Park et al. [22], in which the evaluation of implants was conducted from different directions, a scale analysis (comparing the lowest and highest stability values independently of the direction) yielded significant stability differences in all cases at each follow-up time in the early healing period. However, no significant differences were found when comparing the values between different directional axes. These results suggest that a standardized evaluation method should be established in order to avoid ambiguity when conducting data comparison between studies.

When considering the primary stability at the time of surgery, 10-mm implants showed higher values, but the differences were not significant. This is in accordance with the results of other studies that established no relationship between the variables during this period [13,15,16,17]. Ostman et al. [12] studied ISQ values for 905 implants and observed significant inferior primary stability in longer implants. One possible explanation for this could be that the deeper drilling for longer implants results in an over-prepared implant site, which diminishes the mechanical stability. Conversely, Sim and Lang [10] obtained lower baseline ISQ values for 8-mm implants when compared with 10-mm implants; however, no significant differences could be found, and the authors stipulated that the sample size (n = 16) was slightly small for this purpose. Therefore, based on the results of this paper, the use of longer implants to increase PS is not substantiated.

When analyzing the location variable, it was observed that the ISQ was greater for 10-mm implants than for 11.5-mm implants in the maxilla (*p* = 0.024). As for the mandible, the differences were not significant. When we broke down the regions to look at the anteposterior location, when comparing 10-mm implants with 11.5-mm implants, differences in the anterior mandible could not be evaluated due to the small sample size for the 10-mm implants. Concurrently, 10-mm implants in the anterior maxilla yielded significantly greater values at T0 (*p* = 0.018) and T1 (*p* = 0.031) when compared with 11.5-mm implants.

This difference in primary stability could be due to the intrinsic bone properties in this region. 

Considering the axial axis of the implant, the further we stray from the crestal portion of the maxilla (where there is a relatively thicker cortical compared to other locations) [23], the greater the distance between the cortical plates. Therefore, shorter implants could have a relatively greater bi-cortical anchorage, which, in turn, could alter the primary stability [24]. This hypothesis could also explain the non-significant differences obtained in the posterior area, where the cortical:cancellous bone ratio is lower due to the wider alveolar crest [23]. As such, the ratio of cancellous bone to cortical bone increases. If we consider the overall bone–implant complex rather than the local stiffness at the bone–implant interface [13], the intrinsic properties of the cancellous bone are a greater determining factor than the implant length, per se. Therefore, increased contact with cancellous bone in the posterior area could explain the absence of significant differences [22].

However, this theory could not be corroborated in this study as variables for cortical and cancellous bone were not evaluated. For this reason, future studies should evaluate how the relationship between implants of varying lengths, the cortical:cancellous bone ratio, and the cortical anchorage affect stability values when assessed with RFA. These studies should also take into account the overall bone–implant complex and not just the thickness of the cortical bone on the coronal side of the ridge. In addition, it should be noted that when breaking down measurements for B and L, differences in the anterior area were only significant when measurements were carried out from the buccal side. The reason for this is unknown, as other studies have shown no significant differences between measurements [10].

Regarding SS, we observed that all measurements were affected by increased ISQ values at T1 for both implant lengths. Of the studies reviewed, all presented the same pattern of variability (Table 7).

Concurrently, in the study by Sim and Lang [10], even though an increase in ISQ values at T0 and T1 was observed for both lengths, only shorter implants showed a significant difference between the two follow-up points; a reason for this was not suggested and remains unclear. A similar situation was found in the study carried out by Calvo-Guirado et al. [25], where significant differences were found for short implants only across the different follow-up points.

In terms of percentage, we observed an increase in ISQ values after three months for 10-mm implants in comparison with 11.5-mm implants, and both lengths demonstrated significant differences in terms of stability compared to the time of surgery. Taking these results into consideration, an increase in SS values, in comparison to PS values, can be expected, especially for shorter lengths. In the study by Gómez-Polo et al. [15], irrespective of the bone type and length, implants enjoyed progressive stabilization of the ISQ over the first eight weeks, with values converging at a mean value of 75. The authors hypothesized that this could be due to the fact that gradual growth of bone during osseointegration compensates for any differences in mechanical anchorage. This result differs from the results obtained in our study, where no such compensation took place and significantly greater values were obtained for 10-mm compared to 11.5-mm implants. Furthermore, mean stability values did not converge over the follow-up periods.

Regarding post-functional implant loading, a significant unexpected decrease in ISQ values was observed for both lengths. This differed from the results of a retrospective clinical study published by Brizuela-Velasco and Chávarri-Prado [26], which showed that the functional loading of implants, especially during early stages, increased stability values: ISQ increased by a mean of 5.24% after 6 months and 3.24% after 12 months, while values for implants loaded immediately were significantly greater than those for implants with delayed rehabilitation. In an in vitro study, Kim, Jacobson, and Nathanson [27] demonstrated that provisional cement-retained implant crowns transferred less stress to the implant fixture and supporting structure than screw-retained crowns when vertical forces were applied. This could result in reduced biomechanical stimulation of the implant and surrounding marginal bone, which, in turn, could lead to decreased bone apposition and lowered biological stability over time [28]. We have yet to determine whether the temporary material that was used to fix the crowns in the present study had an influence on SS after implant loading. This hypothesis needs to be evaluated in future in vivo studies. However, besides the decrease in stability, it is important to note that after three months, the differences between lengths disappeared. This fact is in accordance with the results of other authors, who established that irrespective of implant characteristics, ISQ values tend to stabilize over time [21].

There has been controversy regarding attempts to establish the relationship between implant stability measured by RFA and the bone–implant interface, as some ex vivo studies failed to find a correlation between histomorphometrical 2-D data and ISQ values, indicating that the BIC degree does not necessarily reflect the stiffness of the surrounding tissue [29]. Considering this, the assessment that the variation of stability values over time is related to changes in the bone–implant interface could not be scientifically proven. Using ligature-induced peri-implantitis models on Beagle dogs, Monje et al. [30] demonstrated a consistent negative correlation between ISQ measured by RFA and marginal bone loss over time, but they expressed doubt about the clinical relevance of using RFA as a diagnostic tool alone, as even though a statistical relationship was present, the implant stability values remained relatively high over the course of the study. However, the same and other authors agree on the fact that this lack of correlation could have been due to the use of inappropriate methods, citing the fact that a few histological sections from 2-D BIC models cannot represent the complete bone–implant interface; therefore, the analysis of 3-D models in relation to RFA measurements should be more representative [29].

In the work published by Calvo-Guirado et al. [25], after three months of implant placement, all patients showed an increase, followed by a stabilization of RFA values for both 4- and 10-mm implants, and no significant differences were detected between implant lengths at any of the follow-up points. In addition, a significant increase in ISQ was found for 4-mm implants over time. Analyzing this and other variables, the authors concluded that extra-short implants are a feasible treatment option, with radiographic and clinical success rates similar to longer implants. Nonetheless, certain parameters previously discussed should be taken into consideration before comparing the ISQ results among studies. In the former, the selected patients presented atrophic edentulous mandibles, which display greater cortical plates when compared to the dentate variety [23]. In the latter, complete or partial dentitions were used as prosthetic elements for full arch rehabilitations, which made it difficult to predict the significant loading of the implants during function [26].

When comparing stability values between 12 months and the time of surgery, clinically significant differences were found for both lengths; nonetheless, secondary stability in shorter implants showed a relative increase in ISQ values in the months following implant placement when compared with implants of a longer length. Altogether, the results of this study are in accordance with those of other studies [15,17], and suggest that shorter implants have sufficient stability during the early healing period and during the first six months of function. Future studies should investigate the factors contributing to these variations in stability and length during longer periods and how their quantification affects stability values when assessed with RFA.

## 5. Conclusions

Within the limits of the present study, the resonance-frequency analysis conducted at different times indicated that, as far as length is concerned, 10-mm implants have higher ISQ values than 11.5-mm implants over time and greater stability values at three months following implant insertion in partially edentulous patients with shorter implants on single restorations.

Additionally, concerning the locations studied, significant differences were found in the maxilla, with 10-mm implants obtaining higher values than 11.5-mm implants. Furthermore, when analyzing average ISQ values, longer implants showed a lower level of stability in women.

## Figures and Tables

**Table 1:** Comparison of ISQ values for each implant length. Unpaired *t*-test with *p* values.

Implant Length (mm)	Mean (SD)							
	Surgery		After Three Months		After Six Months		After One Year	
	Buccal	Lingual	Buccal	Lingual	Buccal	Lingual	Buccal	Lingual
10	69.53 (11.88)	70.25 (11.80)	72.21 (10.54)	72.00 (10.85)	70.10 (11.16)	69.80 (11.74)	71.57 (11.65)	71.88 (11.23)
11.5	68.50 (10.69)	69.05 (10.87)	70.41 (10.47)	70.00 (10.99)	68.81 (10.40)	69.31 (10.71)	70.45 (9.40)	70.24 (10.44)
*p* value	0.294	0.222	0.047	0.034	0.166	0.617	0.231	0.081

ISQ: implant stability quotient; SD: standard deviation.

**Table 2:** Statistical comparison of absolute ISQ values across the various follow-up points for each implant length represented with *p* values obtained with paired *t*-tests.

Implant Length (mm)	*p* Value			
	T0–T1	T1–T2	T0–T2	T0–T3
10	<0.001	<0.001	0.994	0.002
11.5	<0.001	<0.001	0.864	0.006

ISQ: implant stability quotient; T0: at the time of implant placement; T1: 3 months after implant placement; T2: 6 months after implant placement; T3: 12 months after implant placement.

**Table 3:** Percentage variation of absolute ISQ values across the various follow-up points for each implant length.

Implant Length (mm)	Percentage Variation		
	T0–T1	T1–T2	T2–T3
10	3.02%	−2.94%	2.71%
11.5	2.30%	−2.16%	1.63%

ISQ: implant stability quotient; T0: at the time of implant placement; T1: 3 months after implant placement; T2: 6 months after implant placement; T3: 12 months after implant placement. Significant differences were found between all follow-up points for both lengths (α = 0.05). These levels of statistical significance were determined using the probability parameter (*p*) obtained using a two-tailed student *t*-test for paired data, since they are measurements of the same patient at different times during treatment.

**Table 4:** Comparison of absolute ISQ values for different regions at each follow-up point. Unpaired *t*-tests with *p* values.

Implant Location	Implant Length (mm)	*n*	Mean (SD)			
			Surgery	After Three Months	After Six Months	After One Year
AM	10	17	73.38 (7.69)	76.53 (8.75)	72.29 (12.46)	74.47 (13.66)
AM	11.5	56	67.89 (8.72)	71.21 (6.92)	68.13 (10.81)	70.87 (9.26)
AM	*p* value		0.018	0.031	0.226	0.320
PM	10	76	67.72 (9.97)	70.74 (9.19)	67.60 (9.63)	70.12 (9.48)
PM	11.5	132	67.75 (10.35)	68.98 (10.66)	67.11 (11.81)	68.44 (11.46)
PM	*p* value		0.984	0.238	0.746	0.256

AM: anterior maxilla; PM: posterior maxilla; ISQ: implant stability quotient; SD: standard deviation.

**Table 5:** Comparison of absolute ISQ values for different regions at each follow-up point. Unpaired *t*-tests with *p* values.

Implant Location	Implant Length (mm)	*n*	Mean (SD)			
			Surgery	After Three Months	After Six Months	After One Year
AM	10	1	87.00 (^†^)	85.00 (^†^)	82.00 (^†^)	81.00 (^†^)
AM	11.5	31	68.39 (11.14)	70.45 (9.92)	67.55 (10.67)	69.45 (8.98)
AM	*p* value		*	*	*	*
PM	10	137	70.57 (12.35)	72.26 (11.08)	70.91 (11.49)	72.23 (11.51)
PM	11.5	109	70.58 (11.31)	71.11 (11.86)	71.68 (9.63)	71.98 (9.41)
PM	*p* value		0.997	0.438	0.566	0.885

AM: anterior maxilla; PM: posterior maxilla; ISQ: implant stability quotient; SD: standard deviation. ^†^ No standard deviation could be obtained due to the sample size. * No analysis was performed for different lengths due to the small sample size.

**Table 6:** Comparison of absolute ISQ values between sexes for both lengths.

Implant Length (mm)	Men		Women	
	*n*	Mean (SD)	*n*	Mean (SD)
10	112	70.62 (10.70)	118	71.19 (11.38)
11.5	190	71.21 (9.41)	139	67.14 (11.59)

ISQ: implant stability quotient; SD: standard deviation.

**Table 7:** Comparison of the absolute stability values obtained in various studies for each follow-up point.

Study	Implant Length in mm	Sample Size	Mean (SD)			
			Surgery	3 Months	6 Months	12 Months
Sim and Lang (2010) [10]	8	16	59.8 (21.47)	74.6 (5.94)	-	-
	10	14	70.3 (8.71)	74.8 (4.56)	-	-
Bischof et al. (2004) [13]	8	20	57.7 (7.0)	60.2 (5.1)	-	-
	9	6	57.3 (6.7)	59.2 (9.1)	-	-
	10	24	56.1 (6.1)	61.6 (6.1)	-	-
	11	28	57.9 (5.0)	60.3 (5.9)	-	-
	12	14	57.6 (9.6)	60.6 (6.7)	-	-
	13	14	55.1 (8.2)	57.2 (5.1)	-	-
Calvo-Guirado et al. (2016) [25]	4	40	75.22 (1.23)	78.33 (1.76)	79.65 (0.56)	80.20 (0.44)
	10	20	78.72 (2.13)	81.67 (1.22)	82.45 (0.11)	82.34 (0.67)
